# Above–belowground interactions govern the course and impact of biological invasions

**DOI:** 10.1093/aobpla/plv025

**Published:** 2015-04-08

**Authors:** Mette Vestergård, Regin Rønn, Flemming Ekelund

**Affiliations:** Terrestrial Ecology Section, Department of Biology, University of Copenhagen, Universitetsparken 15, DK-2100 Copenhagen Ø, Denmark

**Keywords:** Belowground–aboveground interactions, context dependency, invasive earthworms, irreversible invasion, reversible invasion, soil biota

## Abstract

Introduction of exotic organisms that subsequently become invasive is considered a serious threat to global biodiversity, and both scientists and nature-conservationists attempt to find explanations and means to meet this challenge. This requires a thorough analysis of the invasion phenomenon in an evolutionary and ecological context; in the case of invasive plants, we must have a major focus on above–belowground interactions. Thus, we discuss different theories that have been proposed to explain the course of invasions through interactions between plants and soil organisms. Further, a thorough analysis of invasion must include a temporal context. Invasions will typically include an initial acute phase, where the invader expands its territory and a later chronic phase where equilibrium is re-established. Many studies fail to make this distinction, which is unfortunate as it makes it impossible to thoroughly understand the invasion of focus. Thus, we claim that invasions fall into two broad categories. Some invasions irreversibly change pools and pathways of matter and energy in the invaded system; even if the abundance of the invader is reduced or it is completely removed, the system will not return to its former state. We use earthworm invasion in North America as a particular conspicuous example of invasive species that irreversibly change ecosystems. However, invasions may also be reversible, where the exotic organism dominates the system for a period, but in the longer term it either disappears, declines or its negative impact decreases. If the fundamental ecosystem structure and flows of energy and matter have not been changed, the system will return to a state not principally different from the original.

## Abstract (precis)

We often consider invasive organisms as one of the most serious threats to global biodiversity, and we want to meet this threat. This, however, requires that we understand invasion in an evolutionary and ecological context; in particular, we must understand interactions between plants and their soil environment. Further, we must understand the temporal context of the invasion, which typically include an initial acute phase and a later chronic phase where equilibrium is reestablished. We claim that invasions fall into two broad categories. Some have irreversible effects, others, however, are reversible, where the exotic organism disappears or its negative impact decreases.

## Introduction

Human introduction—either intentionally or unintentionally—of non-indigenous organisms is often considered one of the most serious threats to existing biodiversity globally (Sala *et al*. 2000). This is because such organisms may become invasive, expand their range dramatically, and thus, severely influence indigenous organisms negatively and cause diversity loss and changes in ecosystems.

Different authors define invasive species differently and the terminology has been heavily debated (Davis and Thompson 2001; Colautti and MacIsaac 2004; Ricciardi and Cohen 2007; Blondel *et al*. 2014). The term ‘invasive species’ is usually synonymized with species established outside their natural range by human action, and that subsequently generate a negative impact on the local ecosystem and the native organisms (Ricciardi and Cohen 2007). The most frequently reported negative impact on the local ecosystem is decreased diversity and decreased abundance of local species. For example, Reinhart and Callaway (2006) define ‘invasive plant species’ as naturalized, non-native species that locally dominate and change relatively diverse existing plant communities into near monocultures. These definitions emphasize the perception that invasive species have negative impacts on their new surroundings. Davis and Thompson (2000, 2001) proposed to restrict the term ‘invasion’ to those situations where the new species have ‘a large impact on the community, ecosystem or economy’. Further, Davis and Thompson (2002) regarded ‘impact’ as a neutral term which should not imply whether the impact is beneficial or harmful to humans. Other researchers have advocated the use of the term ‘invasive’ irrespective of any inference of environmental or economic impact (Richardson *et al*. 2000; Rejmánek *et al*. 2002; Ricciardi and Cohen 2007).

A biological invasion, i.e. when a new species arrives, establishes and spreads in an environment where it did not occur previously, is basically a natural process (Crooks 2002). Hence, there are similarities in the ecological mechanisms operating when plants naturally invade an area during a succession and when an introduced species spreads in a new environment (Davis *et al.* 2001). This has led several authors to argue that we should not see invasive alien species as a specific ecological phenomenon but instead we should try to understand these phenomena in the light of general ecological theory (Davis *et al.* 2011). According to this view, we should focus less on the distinction between non-native and indigenous plants since many native plants behave like invasive non-native plants (Davis *et al*. 2011).

One of the challenges of invasion ecology is to explain why some species can spread rapidly and become dominant in the invaded community while others cannot. For example, only a few of the alien plant species in Britain cause any significant ecological problems (Davis *et al.* 2001). Several authors have attempted to identify properties that make organisms invasive or make ecosystems/habitats susceptible to invasion (Hector *et al.* 2001; Litchman 2010). Clearly, not all potentially invasive organisms will invade any area, and any area susceptible to invasion will not always be invaded; hence, invasion must be understood as an interaction between the invading organism and the invaded ecosystem. In particular, during the last decade, plant interactions with belowground biota have come into focus, and evidence for the significance of these interactions for the establishment and spread of exotic invasive plants is accumulating (Callaway *et al*. 2004; Coats and Rumpho 2014).

It is likewise essential to consider invasion in a temporal context, as its negative impact often decreases over time. Strayer *et al.* (2006), in an excellent review, convincingly argued that studies of invasive species mostly are brief and without temporal context; 40 % of recent studies do not even state the amount of time that had passed since the invasion. According to Strayer *et al*. (2006), invasions have an initial acute phase, immediately after a new species arrives, where the invader expands its territory. This is followed by a chronic phase, after various ecological and evolutionary processes have come into play, where the former invader becomes a non-dominant member of the ecosystem. These processes include genetic changes in the invader, changes in the biological communities in the invaded ecosystem and changes in abiotic conditions in the system.

In this review, we summarize the theoretical framework and results of research on the mechanisms underlying the influence of soil biota on plant invasions. We illustrate that time-dependent alleviation of invasion may be rooted in ecological and/or evolutionary changes of the nature of interactions between invasive species and the belowground biota. Although such natural processes or human intervention can eradicate or diminish their presence in the system, some invasive species fundamentally change the invaded ecosystems, leaving behind a system in another state than the pre-invaded system in terms of, for example, community composition and productivity. We discuss which features of invasive species and invaded systems determine the probability of irreversible impacts on the invaded ecosystem.

## Exotic Establishment: Disrupted Belowground–Aboveground Interactions?

Several hypotheses have been suggested to explain the overwhelming success of invasive exotic plant species, which apply to the acute and chronic phases outlined above. Here, we will focus on hypotheses related to interactions between plants and soil biota, as the success of invading plants at the expense of indigenous plant communities in many cases is governed by interactions with soil organisms (Klironomos 2002; Engelkes *et al*. 2008; Mangla and Callaway 2008; Callaway *et al*. 2011; Maron *et al*. 2014). Native species often suffer reduced growth when grown in the same soil for consecutive generations (negative plant–soil feedback), whereas invasive species are more likely unaffected or positively affected by growing in the same soil for several successive generations (neutral or positive plant–soil feedback, respectively) (Klironomos 2002). Soil sterilization interrupts these feedback effects (Klironomos 2002), which clearly shows that soil biota must play a role. Moreover, several studies suggest that invasive plants experience less negative feedbacks from soil biota in their invasive range than in their native range (Reinhart *et al*. 2003; Callaway *et al*. 2004, 2011; Maron *et al*. 2014). This has prompted formulation of several very interesting hypotheses to explain the biogeographic basis for the differences in biotic feedback mechanisms. The available empirical data are still too scarce to dismiss or accept these hypotheses, but they represent rigorous concepts for future developments of our understanding of the mechanisms and processes that render some plants invasive. In the following, we give an account of the hypotheses and empirical studies addressing these possible mechanisms behind belowground biotic impacts on plant invasions.

### Enemy release belowground

The enemy release hypothesis states that the lack of natural specialized enemies, i.e. herbivores and pathogens, allows the successful invasion of populations introduced to new ranges (Keane and Crawley 2002; Mitchell *et al*. 2006). Only very few studies have tried to identify specific soil organisms that are actually involved. Reinhart *et al*. (2003, 2010), though, showed that soil sterilization had a positive effect on invasive *Prunus serotina* seedlings grown in soil from its home range, but a negative effect on seedlings grown in soil from its non-native invasive range. Interestingly, *Pythium* pathogens from *P. serotina*'s native North American range increased root-rot by 38–462 %, seedling mortality by 80–583 % and reduced biomass production by 19–45 % compared with *Pythium* taxa from the European invasive range (Reinhart *et al*. 2010). Hence, the escape from more virulent North American *Pythium* taxa may explain the uncontrolled spread of the species in Europe.

In contrast, differences in *Pythium* virulence could not explain more negative feedbacks of soil biota from the native than from non-native ranges on *Robinia pseudoacacia* (Callaway *et al*. 2011). Here, removal of organisms larger than ∼20 μm made the remaining soil biota from the invasive range more harmful to *R. pseudoacacia* than the total soil biotic assemblage—actually as harmful as the soil biota from the native range (Callaway *et al*. 2011). Rather than enemy release, this suggests that in the invasive range, soil organisms larger than 20 μm reduce the harmful effects of smaller soil organisms. Hence, we cannot necessarily explain the net effect of the combined soil biota on plant growth through detailed identification and quantification of plant growth promoting and pathogenic soil organisms. We need to also consider that soil organisms that exert a direct influence on plants are also affected by complex interactions with the many different organisms that encompass the total soil biota (e.g. Bjørnlund *et al*. 2006; Rønn *et al*. 2012).

The native European dune grass, *Ammophila arenaria*, has spread vigorously in introduced regions of Tasmania, New Zealand, South Africa and the USA. *Ammophila arenaria* experienced less negative soil-feedback in South African soil than in soil from its native European range (Knevel *et al*. 2004), which, in part, can be explained by the lack of specialist root-feeding nematodes in the introduced ranges (Beckstead and Parker 2003; van der Putten *et al*. 2005). However, in Californian soil, negative soil feedback effects on *A. arenaria* germination and growth were comparable with negative feedback effects in the native range of the species (Beckstead and Parker 2003); hence enemy release cannot fully explain Californian *A. arenaria* invasion. Though the above studies suggest that escape from specialized belowground enemies facilitate plant invasions, we still need more detailed biogeographic comparisons of the presence and incidence of identified soil-borne pathogens and herbivores to seriously evaluate the belowground enemy release hypothesis.

### Novel weapons

According to the novel-weapons hypothesis (Callaway and Aschehoug 2000), invasive plants are successful because they possess inhibitory allelochemicals that are novel in their invasive range. Hence, soil organisms, including soil-borne pathogens, have not adapted to these chemicals. For example, Zhang *et al*. (2009) found that root and rhizome extracts of invasive *Solidago canadensis* reduced growth and pathogenic activity of the two soil pathogens *Pythium ultimum* and *Rhizoctonia solani*, and the marine red alga *Bonnemaisonia hamifera* avoids herbivory in its invasive range due to its content of a specific defence compound that is unknown in native algae from the invaded range (Enge *et al*. 2012). Release of the allelochemical catechin by *Centaurea stoebe* reduces nitrification, and this effect was much more pronounced in soil from *C. stoebe*'s invasive range than in its native soil (Thorpe and Callaway 2011). This suggests that nitrifying bacteria in the invasive range were more susceptible to catechin than nitrifying bacteria with a long evolutionary history of catechin exposure. There is still limited empirical evidence that the possession of defence compounds novel to soil-borne enemies in the introduced range can explain plant invasions. However, as summarized in the following section, novel chemicals of an introduced plant species may inhibit mycorrhizal fungi.

### Suppression of mutualists

Approximately 80 % of angiosperms associate with one or more mycorrhizal fungal species (de Boer *et al*. 2005). This association is often mutually beneficial to host plant and fungus (Klironomos 2003). Hence, disruption of the symbiotic association can retard host plant competitive ability. *Alliaria petiolata*, native to Europe, lacks mycorrhiza like other Brassicaceae. *Alliaria petiolata* is invasive in North American forest understories, and generally causes more negative soil feedbacks for North American than European understory plants (Callaway *et al*. 2008). Further, arbuscular mycorrhizal fungi (AMF) spore densities, spore viability and spore infectivity were reduced in North American soils, which had hosted *A. petiolata*, whereas these effects were absent in European soils. *Alliaria petiolata* invasions in North America are thus likely governed by flavonoids released from *A. petiolata* roots that are effectively killing mycorrhizal fungi in North America, but not in the European native range, where *A. petiolata* and fungi have co-existed on a long evolutionary timescale (Stinson *et al*. 2006; Callaway *et al*. 2008). Hence, lack of adaptation to novel weapons released by invasive plants can, at least partly, explain the suppression of mutualists. Similarly, arbuscular mycorrhizal colonization, nutrient uptake and growth of *Elymus elymoides*, native to North America, were reduced when it was sown in North American soil with the invasive *Bromus tectorum*, which has its origin in Eurasia (Owen *et al*. 2013).

Ectomycorrhizal mutualist associations may also be disrupted by exotic invasive plant species. For instance, invasive *Fallopia* × *bohemica* reduced ectomycorrhizal colonization of native *Tsuga* seedlings by 64 % (Urgenson *et al*. 2012), and both arbucular mycorrhizal and ectomycorrhizal colonization of *Populus fremontii* decreased when *P. fremontii* grew in the vicinity of invasive *Tamarix* sp. (Meinhardt and Gehring 2012). Further, Grove *et al*. (2012) found that allelopathic compounds of invasive *Cytisus scoparius* reduced growth of native Douglas Fir (*Pseudotsuga menziesii*) concomitant with reduced ectomycorrhizal abundance. Since mycorrhizal associations can be a major determinant of local plant species diversity (Klironomos 2003), these interactions likely play an important role for the probability of the invading plant to establish and persist in the invaded ecosystem.

### Accumulation of local pathogens

Eppinga *et al*. (2006) suggested ‘The accumulation of local pathogens hypothesis’ as enemy release did not satisfactorily explain *A. arenaria* invasion of Californian dune systems. They hypothesized that *A. arenaria* in its non-native range accumulates generalist soil pathogens that are relatively more harmful to native species than to *A. arenaria* itself. Empirical evidence for this hypothesis is scarce, but *Chromolaena odorata* invasion throughout the Old World tropics and subtropics may be rooted in *C. odorata* stimulation of *Fusarium* strains that are pathogenic to indigenous plants in the invaded area (Mangla and Callaway 2008).

## Invasions Are Context-Dependent

In accordance with the large number of theories that try to explain biological invasions, most likely, the phenomenon has different causes and consequently must be explained differently in different cases. Further, the strength and direction of interactions between introduced and native species, as well as between above- and belowground biota depends on both biotic and abiotic features of the system to which an alien species is dispersed.

### Susceptibility to allelochemicals

The probability that an alien plant with allelopathic activity will become invasive depends on the susceptibility to the allelochemicals of the indigenous plant and soil community. The susceptibility to allelochemicals from the invasive *Lonicera maackii* varied between plant species and, for some plants, soil organisms reduced the negative impact of *L. maackii* allelochemicals, whereas for other plants, activity of soil organisms enhanced negative effects imposed by *L. maackii* allelochemicals (Bauer *et al*. 2012). In some cases, microbial degradation of allelochemicals thus eliminates or reduces their effects (Inderjit 2005; Kaur *et al*. 2009; Inderjit *et al*. 2010), whereas in others, partial microbial decomposition of allelochemicals may produce derivatives exerting stronger allelopathic effects than the intact allelochemical (Inderjit 2005).

The composition of the soil microbial community can also determine the extent to which an allelochemical is degraded and hence determine its actual effect. For instance, the phytotoxin juglone, released by Black Walnut (*Juglans nigra*), is broken down by *Pseudomonas putida*, and it appears that the accumulation of juglone in soils under Black Walnut depends on the presence or absence of *P. putida* (Inderjit 2005). Further, the abiotic environment of a potentially invaded system can also determine the impact of putative allelochemicals. For instance, soil metal (Pollock *et al*. 2009) and organic matter content (Loffredo *et al*. 2005; Tharayil *et al*. 2006) can be decisive for the persistence and activity of allelochemicals.

### Composition of belowground communities

The composition of the belowground communities may affect success of potential invaders via soil feedback in more complex ways as discussed above (Klironomos 2002; Callaway *et al*. 2011; Parepa *et al*. 2013). For example, a pot experiment demonstrated that the invasion success of *Bidens pilosa* in a plant community with five Hawaiian species depended on which species of AMF were present (Stampe and Daehler 2003). Likewise, the invasive success of *Anthemis cotula* is related to the composition of the AMF community (Shah *et al*. 2008). In experimental plant communities, the chance of establishment of invaders, notably *Erigeron canadensis* and *Taraxacum officinale*, was clearly reduced when *Leucanthemum vulgare* was part of the existing plant community (van Ruijven *et al*. 2003). As *L. vulgare* did not dominate any of the experimental plant communities, invasion resistance could not be explained by dominance. But plant communities with *L. vulgare* had high incidences of plant feeding nematodes, thus the authors hypothesized that the accumulation of particular plant feeding nematodes, perhaps combined with nematode-assisted virus transmission, reduced the establishment of invaders. This example illustrates the potential significance of complex interactions between individual plant species, components of the belowground biota and establishment of competing plant species, and underpins that the invasive success of an introduced species or the invasibility of a system can depend on the combination of organism interactions.

### Disturbance

Invasion biology of macro-organisms is difficult to approach experimentally because a complete invasion sequence normally takes many years (Strayer *et al.* 2006), and research grants normally will not have that duration. Therefore, it may be attractive to examine biological invasions experimentally using microorganisms. As an example, we used laboratory soil microcosms to study invasion and establishment success of *Pseudomonas fluorescens* during a 42-day period (Liu *et al*. 2012). We used soil heating to create a disturbance gradient, and hypothesized that increased disturbance would facilitate invasion, which was confirmed by the experiments. This suggests that the key factors associated with the heating disturbance that explain the enhanced invasion success are increased carbon substrate availability and reduced diversity, and thus, competition- and predation-release. In a second experiment, we therefore separated the effects of increased carbon availability and decreased diversity. Here, we demonstrated that the effect of the indigenous soil community on bacterial invasion was stronger than that of resource availability. In particular, introduced bacteria established better in a long-term perspective at lower diversity and predation pressure.

Disturbance also proved conducive for the establishment of introduced plant species in an experimental set up to assess the relative importance of plant species traits and extrinsic factors on the establishment of introduced plants in grassland systems (Kempel *et al*. 2013). Moreover, the relationship between some plant traits and establishment success depended on disturbance regime. For instance, hypocotyl elongation in response to shading was a disadvantage for establishment in disturbed, but not in undisturbed systems. Further, the influence of both environmental factors and plant traits on establishment success depended on time since introduction. Hence, whereas seed mass played a large role for early establishment, the persistence with time depended on traits relevant for biotic interactions such as resistance against generalist herbivores and response to competition (Kempel *et al*. 2013).

Disturbed systems thus appear relatively more prone to invasion than undisturbed systems (González-Moreno *et al.* 2014; Houseman *et al*. 2014), but the larger impact of earthworm invasions in undisturbed North American forests (discussed below) than in systems that have been ploughed (Bohlén *et al*. 2004*a*) illustrates that the impacts imposed by an invasive organism also depends on the disturbance legacy of the invaded system. Thus, invasiveness of a particular introduced species is determined not only by features of the introduced species, but emerges as the combination of traits of the introduced organism, identity and diversity of the organisms residing in the potentially invaded system as well as physico-chemical properties of this system.

## Reversible and Irreversible Invasions

Invasive species can affect the system they invade in several ways, and the magnitude of their environmental impact varies greatly (Blackburn *et al*. 2014; Jeschke *et al*. 2014). Some invasions are transient and their impact decreases with time (Strayer *et al*. 2006), others have long-term effects and in some cases these effects are irreversible, even if the invasive organism is totally eradicated. Below we illustrate how evolutionary and ecological processes in some cases alleviate the impacts of invasions, whereas some invasions lead to long-term, sometimes irreversible, fundamental ecosystem changes.

### Alleviation of invasion by re-establishment of biotic control mechanisms

Some invasions certainly follow the temporal pattern of an initial acute phase after which the abundance of the exotic invader declines and it becomes a non-dominant member of the ecosystem. To the extent that plant invasions are facilitated by disrupted belowground control mechanisms (see above), a re-establishment of these mechanisms will in some cases lead to decreased abundance and/or impact of the invader. *Heracleum mantegazzianum* is the most well-known exotic invasive plant species in many parts of Europe. It is native to the Caucasus, where it is a non-dominant member of meadow and forest clearing communities (Nielsen *et al*. 2004). Since it was introduced to Western Europe in the 19th century, it has spread vigorously and is now considered a problem throughout Europe (Nielsen 2007). However, the negative impacts of *H. mantegazzianum* on invaded ecosystems may actually only be temporary. Hence, for grassland sites invaded by *H. mantegazzianum* for different time periods, the *H. mantegazzianum* cover declined with length of time since the sites were invaded, and initial negative effects of *H. mantegazzianum* on native species richness and productivity were alleviated after 30 years of invasion (Dostál *et al*. 2013). Pot experiments suggested that the *H. mantegazzianum*-decline over time could in part be explained by the accumulation of soil pathogens with a negative impact on *H. mantegazzianum* fitness, or alternatively, evolution of higher soil pathogenicity to *H. mantegazzianum* in soils with a long legacy of *H. mantegazzianum* exposure (Dostál *et al*. 2013). Similarly, Diez *et al*. (2010) reported that for 12 plant species, considered invasive in New Zealand, the strength of negative plant–soil feedbacks increased with time since they established. They hypothesized an accumulation of belowground enemies to be the explanation.

Based on data compiled on fungal and viral pathogen species richness for 124 plant species of European origin introduced to North America, Mitchell *et al.* (2010) demonstrated that plants introduced 400 years ago hosted six times as many pathogens as plant species that were only introduced 40 years ago. Hence, the accumulation of pathogens in the introduced range may be a relatively slow process spanning decades and even centuries. Mitchell *et al.* (2010) argue that the cumulative probability of pathogen accumulation increases with time, because (i) the probability of co-introduction or delayed introduction of pathogens from the exotic species’ home range increases with time as does (ii) the probability of pathogen transfer from native plant species and (iii), as the introduced species expands with time, it will get exposed to increasing numbers of alternative host plants, abiotic conditions and habitat types which can be expected to support different pathogens.

Further, it is likely that introduced plants also experience time-dependent increased herbivore or pathogen pressure, if native belowground herbivores or pathogens evolve features that allow them to control the exotic plant. This mechanism has been demonstrated aboveground, where the native Australian soapberry bug (*Leptocoris tagalicus*) evolved 5–10 % longer mouthparts, which allowed them to attack larger fruits of the forest-invading exotic balloon vine (*Cardiospermum grandiflorum*) (Carroll *et al*. 2005).

### Reduced plant impact on soil communities

The negative impact on soil communities of allellochemicals released by invasive plants can decrease with time due to changes in plant production of allellochemicals. The European Garlic Mustard, *A. petiolata* (discussed above), releases allelochemicals with negative effects on seed germination of neighbouring plants (Rodgers *et al*. 2008) and antimycorrhizal effects (Stinson *et al*. 2006; Callaway *et al*. 2008). In recently invaded sites *A. petiolata* reduced belowground microbial richness. However, soil microbial richness recovered after long-term exposure to the plant (Lankau *et al*. 2009, Lankau 2011). Hence, it appears that the suppressive effect on mutualists and other soil-dwelling microorganisms can be a transient phenomenon during *A. petiolata* invasions. A likely explanation could be that the *A. petiolata* production of glucosinolates decreased with time since invasion (Lankau *et al*. 2009). Further, the increase-rates of *A. petiolata*-cover decreased with time since invasion, and concomitantly the cover of native woody species increased with increasing rates during the surveyed chrono-sequence (Lankau *et al*. 2009). Lankau *et al*. (2009) suggested that what appears as decreasing ability to compete with native species during their expansion at a given location can be explained by evolutionary changes within *A. petiolata* populations; hence, old populations produced less glucosinolates, which are toxic to other plant species than populations who had recently invaded a new area. We notice, though, that this line of argumentation may suffer from the shortcoming that new populations probably have spread from old; a down-regulation of genes involved in glucosinolate production may be more likely.

### Long-term impacts of plant invasions

Plant species that cause a significant change in the invaded ecosystem have been termed ‘transformer species’ (Richardson *et al*. 2000; Sheppard *et al*. 2010). Richardson *et al*. (2000) suggested eight categories of transformer species: (i) excessive users of resources, (ii) donors of limiting resources (e.g. nitrogen fixers), (iii) plants that promote or suppress fire, (iv) sand stabilizers, (v) erosion promoters, (vi) sediment stabilizers on intertidal mudflats, (vii) litter accumulators and (viii) salt accumulators. For example, nutrient losses associated with plant invasions may have severe and irreversible consequences for the invaded system. Invasion of the tall and dense Gamba-grass (*Andropygon gayanus*) in Northern Australian savannas has provided fuel loads, which have increased fire intensity and thus caused increased fire-mediated nitrogen losses of these already nutrient poor ecosystems (Rossiter-Rachor *et al*. 2008). Invasion of *Cocos nucifera* at the Palmyra atoll indirectly led to ecosystem nutrient depletion, because birds avoid nesting or roosting in *C. nucifera* (Young *et al*. 2010). Consequently, bird-mediated nutrient import from the marine environment to the terrestrial ecosystem is disrupted with cascading ecosystem effects such as reduced soil nutrient availability, leaf nutrient content and palatability and herbivory (Young *et al*. 2010).

Conversely, invasion by nitrogen-fixing plants in nitrogen-limited systems dramatically enhances soil nitrogen availability (Vitousek *et al*. 1987; Rice *et al.* 2004), which has long-term consequences for primary production and plant communities (Maron and Connors 1996; Marchante *et al*. 2011; Benesperi *et al.* 2012). For instance, N-fixing *Acacia longifolia* invasions of Portuguese coastal dunes enhanced litter C and N accumulation by 3.5 and 5 times, respectively, and dramatically increased soil cation content over a >20 year period, which significantly enriched the soil of this low-productivity system and altered belowground microbial activities and N cycling (Marchante *et al.* 2008). Although removal of exotic N-fixing *R. pseudoacacia* from an inland sand barren system reduced soil N concentrations and total net N-mineralization rates to pre-invasion levels already after 2 years, nitrification rates remained 3–34 times higher in areas from which *R. pseudoacacia* were removed compared with uninvaded areas (Malcolm *et al*. 2008).

### Irreversible effects: ecosystem engineers

Earthworms are particularly good examples of invasive ecosystem engineers; i.e. organisms which modify their habitat by changing the physical state of the environment (Jones *et al*. 1994; Crooks 2002) and cause dramatic changes in the invaded ecosystem. Earthworms play a very essential role in the breakdown of organic matter and may significantly change basic soil properties (Edwards and Bohlén 1996). North America harbours more than 100 species of native earthworms but the earthworm fauna was strongly affected by the Pleistocene glaciations and in most of Canada and the northern part of the USA there are virtually no native earthworms (Hendrix and Bohlén 2002).

However, since the European settlement, lumbricid earthworms have been colonizing the northern hardwood forests and they are now widespread in the region with marked consequences for the forest ecosystems (Bohlén *et al*. 2004*b*; Frelich *et al*. 2006). Comparisons of areas with and without earthworms, and along leading edges of earthworm invasion, indicate that earthworm invasion affects many soil properties. The forest floor in unaffected earthworm-free forests is usually characterized by a well-developed organic O-horizon but earthworm invasion leads to reduced O-horizons and increased thickness of the A-horizon (Alban and Berry 1994; Hale *et al*. 2005; Eisenhauer *et al*. 2007). Incorporation of organic material into deeper soil layers may affect cycling of C, N and P (Bohlén *et al*. 2004*b*; Eisenhauer *et al*. 2007) and lead to reduced availability of N and P for plants with shallow root systems (Hale *et al*. 2005; Frelich *et al*. 2006). Studies from New Zealand demonstrate that the changes in soil properties brought about by the presence of European endogeic earthworms increase productivity of pastures significantly (Stockdill 1982). The changes in soil properties also change understory plant communities (Frelich *et al*. 2006; Hale *et al*. 2006). For example, Hale *et al*. (2006) found a change in community composition as well as reduced plant diversity in the presence of earthworms. Part of the changes in plant communities may be mediated through effects on the fungal community, through reductions in mycorrhizal colonization (Lawrence *et al*. 2003). Hence, earthworm invasion can favour non-mycorrhizal plants, such as e.g. the invasive herb *A. petiolata* (Bohlén *et al*. 2004*b*).

The effect of earthworms on the ecosystem also depends on land-use history. Hence, the effect is less marked in areas which have been previously cultivated compared with undisturbed forests (Bohlén *et al*. 2004*a*), probably because earthworms affect soil similarly to ploughing (Frelich *et al*. 2006). Recovery of soils from cultivation takes several centuries (Frelich *et al*. 2006) and the effect of earthworms will probably have equally long-lasting effects. Hence, earthworms are an example of an invasive organism with dramatic effects in the ecosystem. Even if it were somehow possible to remove invasive earthworm species from North America, the effects on the system would still be seen for many centuries to come.

### Can we predict the risk of long-term or irreversible impacts?

Efficient strategies for management of invasive species depend on our ability to predict which invaders would have the largest impact and whether or not the impact is reversible. Presently, we do not even have qualified accounts of long-term impacts of invasive species. As outlined above, we know of cases where invasive species have caused long-term changes of their invaded ecosystems, but whether plant invasions in general pose long-term risks to ecosystems remains an open question that needs to be addressed. Further, the large context dependency of both invasion events, the magnitude of impact on the invaded systems as well as the extent to which the impacts are reversible or irreversible makes it difficult to devise a generalized scheme of prediction.

Some features do appear to render ecosystems vulnerable to invasion by alien species, though. It thus appears that disturbed ecosystems are more susceptible to invasion. As demonstrated for microbial systems, this increased invasion risk can be related to the loss of functions, i.e. consumers of invasive species, in the disturbed system.

Likewise, the impact of an invasive species also appears to be related to functioning. Hence, the probability that an invasive species causes long-term or even irreversible changes of the invaded ecosystem increases for invasive species that occupy functional roles that are new to the invaded system. Ecosystem transformers that changes pools of energy and elements, e.g. N-fixing plants in N-limited systems, or ecosystem engineers, i.e. earthworms, that physically re-organize the invaded ecosystem are therefore more likely to cause long-term or irreversible impacts on the invaded ecosystem.

We thus emphasize that long-term impacts of ecosystem transformers and engineers depend on the functionality of the invasive species in relation to the functions already represented in the invaded system. In other words, we suggest that the probability that an invasive species will change the invaded system irreversibly decreases with the number of niches and numbers of functions already represented in the system. As such, systems with a high degree of isolation in space and time such as small, isolated islands should be considered more at risk of long-term impacts.

## Conclusions

We notice that invasions fall into two broad categories. (i) Irreversible, i.e. the invasion fundamentally, and irreversibly, changes pools and pathways of matter and energy in the system. Here we emphasize that significant changes in community structure, such as e.g. permanent loss of species, also fall into this category. If the abundance of the invader is reduced or the invader is even completely removed, the system will not return to its former state. (ii) Reversible: the exotic organism dominates the system for a period, but in a longer perspective it either disappears, declines or its negative impact decreases. If the fundamental ecosystem structure and flows of energy and matter have not been changed, the system will return to a state not principally different from the original.

It is becoming increasingly clear that the composition of belowground biota is a crucial ecosystem feature to consider if we are to understand the mechanisms that facilitate or reduce invasion of alien plant species. For instance, the lack of natural belowground enemies may pave the way for plant invasions, but this hypothesis must be supported by more direct evidence of biogeographic differences in the distribution of identified belowground pathogens and herbivores. However, here we stress that not only direct interactions between potentially invasive plants and closely affiliated soil organisms (herbivores, pathogens or symbionts) must be taken into account. Rather, both the plant roots and its herbivores, pathogens and symbionts reside within a complex and highly diverse belowground biota, where a multitude of interactions occur, which can, indirectly, affect the direct impacts of specific soil organisms and the plant, e.g. via predation of or competition with the plant-affiliated organism. Thus, the course of invasions is context dependent; i.e. the mechanisms at play depend on features of both the invasive species and the invaded ecosystem.

## Sources of Funding

M.V., R.R. and F.E. were all funded by The Danish Council for Independent Research (OP-RICE-ING, DFF—4002-00274) and by The Danish Council for Strategic Research (ASHBACK, DSF—12-132655).

## Contributions by the Authors

M.V., R.R. and F.E. contributed equally to the manuscript.

## Conflicts of Interest Statement

None declared.
